# Feasibility of magnetic bead technology for concentration of mycobacteria in sputum prior to fluorescence microscopy

**DOI:** 10.1186/1471-2334-11-125

**Published:** 2011-05-13

**Authors:** Heidi Albert, Patrick J Ademun, George Lukyamuzi, Barnabas Nyesiga, Yukari Manabe, Moses Joloba, Stuart Wilson, Mark D Perkins

**Affiliations:** 1 FIND, Foundation for Innovative New Diagnostics, Kampala, Uganda; 2 Infectious Diseases Institute, Mulago Hospital, Kampala, Uganda; 3 Department of Medical Microbiology, Makerere University, Kampala, Uganda; 4 National Tuberculosis Reference Laboratory, Wandegaya, Kampala, Uganda; 5 Microsens Medtech Ltd., London, UK; 6 FIND, Foundation for Innovative New Diagnostics, Geneva, Switzerland

## Abstract

**Background:**

Direct sputum smear microscopy is the mainstay of TB diagnosis in most low and middle income countries, and is highly specific for *Mycobacterium tuberculosis *in such settings. However it is limited by low sensitivity, particularly in HIV co-infected patients. Concentration by centrifugation has been reported to be more sensitive than direct smear preparation, but is only suitable for referral laboratories. Simpler concentration methods that could be applied in peripheral laboratories are urgently needed.

**Methods:**

We evaluated the feasibility of an early prototype ligand-coated magnetic bead technology to concentrate *M. tuberculosis *prior to detection by LED-based fluorescence microscopy compared with direct Ziehl-Neelsen microscopy and direct and concentrated fluorescence microscopy in a reference laboratory in Kampala, Uganda. Results were compared with MGIT 960 liquid culture and Lowenstein-Jensen culture.

**Results:**

Compared to culture, concentrated FM had significantly higher sensitivity than direct ZN (74.8% and 51.4%), magnetic bead-FM (65.4%) and direct FM (58.9%). The sensitivity of magnetic bead FM was significantly higher than direct ZN (p < 0.001) but not significantly higher than direct FM (p = 0.210). The specificity of magnetic bead FM and concentrated FM was significantly lower than direct ZN (88.6%, 94.3% and 98.9% respectively) and direct FM (99.4%). There was no significant difference in specificity between magnetic bead FM and concentrated FM. Allowing for blinded resolution of discrepant results, the specificity of magnetic bead FM increased to 93.1%. Direct microscopy was simpler than concentrated FM and Magnetic bead FM which both had a similar number of steps.

**Conclusion:**

The sensitivity of the early prototype magnetic bead FM was lower than concentrated FM, similar to direct FM, and significantly higher than direct ZN. Both magnetic bead and concentration by centrifugation led to reduced specificity compared with the direct smear methods. Some magnetic bead FM false positive results were not easily explained and should be further investigated. The prototype version of the magnetic bead procedure tested here was of similar complexity to concentration by centrifugation. As such, if the sensitivity of the magnetic bead FM could be improved in future versions of the technology, this may offer a viable alternative to centrifugation.

## Background

Direct sputum smear microscopy is the mainstay of TB diagnosis in most low and middle income countries, where 95% of TB cases and 98% of deaths associated with TB occur [1]. This method is rapid and inexpensive and highly specific for *Mycobacterium tuberculosis *in such settings. Furthermore, microscopy is a multi-disease platform that can be used for diagnosis of a number of diseases of public health importance. However the main limitation of the method is its low sensitivity in programmatic settings, particularly in HIV co-infected patients [2].

Various physical and chemical sputum processing methods have been identified which can improve the sensitivity of microscopy. A recent systematic review ^1 ^concluded that processing by a several chemical procedures, followed by centrifugation or overnight sedimentation, were more sensitive than direct microscopy, and that specificity was similar [3]. However, many such methods require expensive centrifuges or reagents which would add substantially to the cost and complexity of performing smear microscopy. Fluorescence microscopy (FM) gives an average increase of 10% sensitivity over the ZN method, while retaining specificity [4], a finding which has had little relevance in disease endemic countries until the recent development of inexpensive fluorescence microscopes illuminated by light emitting diodes (LEDs).

Immunomagnetic capture is used in a number of platforms as an alternative to filtration or centrifugation to concentrate bacteria in clinical specimens. This method has been applied to concentration of mycobacteria in cerebrospinal fluid [5] and in environmental samples [6], with polymerase chain reaction and culture as detection end-points. A recent study reported encouraging performance of ligand-coated magnetic beads in combination with FM, in detection of *M. tuberculosis *in a panel of frozen sputum samples, with good correlation reported between magnetic bead concentration and centrifugation [7].

This study evaluated the feasibility of using an early prototype ligand-coated magnetic bead technology to concentrate *M. tuberculosis *prior to detection by LED-based fluorescence microscopy (magnetic bead FM). The magnetic beads are coated with a chemical ligand that selectively binds mycobacteria (including *M. tuberculosis *complex and non-tuberculous mycobacteria) and does not bind many other bacterial species. The ligand is not affected by pH, which allows the TB to be captured directly from sputum decontaminated using sodium hydroxide. The method does not require a centrifuge or other major equipment and so may be applicable to peripheral laboratories [7].

In this study, the performance of an early prototype ligand-coated magnetic bead processing method (Microsens Medtech Ltd., London, United Kingdom), used in conjunction with FM, was compared to direct ZN microscopy, direct LED FM microscopy, and LED FM microscopy after NALC-NaOH concentration. The applicability of the magnetic bead technology in high burden settings was also assessed in terms of complexity and hands-on time compared with standard methods. The cumulative yield of each method with varying examination times was also investigated to assess the reading time required for optimal diagnostic yield for each method.

## Methods

### Study setting and design

Leftover portions of sputum specimens submitted for routine tuberculosis diagnostic investigations were collected from the Microbiology Laboratory at Mulago National Referral Hospital for inclusion in this study. Sputum specimens were collected from TB suspects; follow up specimens for the purpose of treatment monitoring were excluded from this study.

### Laboratory testing

All sputum specimens with at least 2 ml volume were included in this study. Direct FM was performed at the Mulago Hospital Microbiology Laboratory. Specimens were refrigerated immediately after preparation of routine direct FM smears and transported to the FIND laboratory on the day of collection.

An additional direct sputum smear was stained by Ziehl-Neelsen method, and examined according to World Health Organisation (WHO)/International Union Against Tuberculosis and Lung Disease (IUATLD) guidelines [8]. Grading charts were used for all smear readings. Quantification of smear results was as follows: 1-9 acid-fast bacilli (AFBs) per 100 fields (report exact count); 10-99 AFBs per 100 fields (1+); 1-10 per field (2+) and >10 per field (3+).

Specimens were then homogenized and split for concentration fluorescent microscopy using either magnetic bead or conventional NALC-NaOH decontamination. Following aseptic addition of 6-8 sterile 3 mm diameter glass beads, specimens were vortexed for 30 seconds to homogenize the specimens. Two 1 ml portions of homogenized sputum were transferred to separate sterile tubes using a sterile transfer pipette. Tubes were coded to ensure that the technicians performing and reading the results of each method were not aware of the results of alternative reads. On alternate days the first portion was transferred either to the tube for magnetic bead processing or to the decontamination/centrifugation tube to reduce sampling bias. Culture results were also interpreted independently from the smear results. Un-blinding was undertaken at the end of the study at the time of data analysis. All manipulations with potentially infectious clinical specimens were performed in a Class II safety cabinet in a BSL 3 Laboratory.

### Standard decontamination and centrifugation method

One tube was decontaminated by standard N-acetyl cysteine (NALC)-sodium hydroxide (NaOH) procedure according to standard methodology (1.5% final concentration NaOH) [9]. Centrifugation was carried out at 3000 *g *for 20 minutes, using sealed aerosol-free buckets, which were only opened inside the biosafety cabinet. Following buffering and centrifugation 0.1 ml phosphate buffer pH6.8 was added to re-suspend the pellet.

A smear was prepared from 1 drop (approximately 40 μl) of the decontaminated suspension and allowed to air dry. Slides were stained with Auramine O and read according to WHO/IUTALD guidelines [8]. Fluorescence microscopy was performed using Primo Star iLED microscope (Carl Zeiss MicroImaging GmbH) at 400× magnification. For quantification of fluorescent stained smears, 1-19 AFBs per length (report exact count); 20-199 AFBs per length (1+); 5-50 AFBs on average per field (2+); >50 per field on average (3+).

Following smear preparation, an additional 0.5 ml phosphate buffer was added to the processed sputum suspension and mixed using a vortex mixer. Of this, 0.5 ml was used to inoculate MGIT culture and 0.1 ml to inoculate a Lowenstein-Jensen medium slant. Positive cultures were identified as *M. tuberculosis *species using the Capilia TB Neo assay (Tauns, Numazu, Japan).

### Magnetic bead processing method

**Figure 1:**
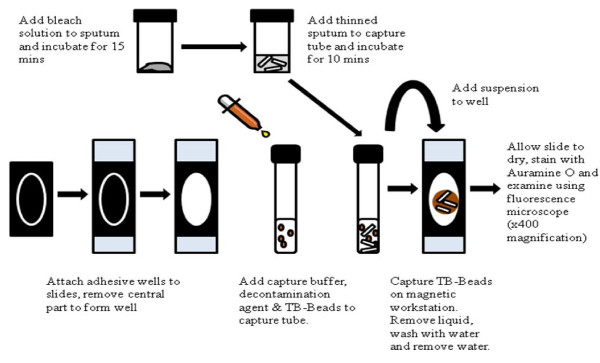
**Diagram of Magnetic bead procedure**.

One tube was processed according to the prototype magnetic bead processing protocol (Microsens Medtech Ltd., London, UK) (Figure 1).

The following components were provided by the manufacturer: bleach tablets (for thinning solution), adhesive wells, capture buffer, ligand-coated magnetic beads and magnetic workstation.

A master thinning solution (10X concentration) was prepared by dissolving 1 bleach tablet in 100 ml distilled water. On a daily basis, this master solution was diluted 1:10 in distilled water to make a working concentration. Decontamination agent consisted of 4% N-acetyl cysteine and 6% sodium hydroxide (Sigma Chemical Co.) dissolved in distilled water. Decontamination agent was prepared daily.

**Figure 2:**
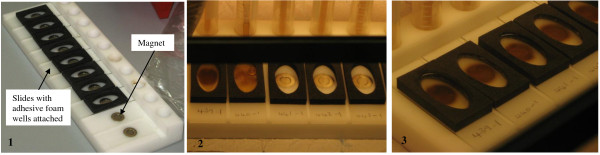
**Steps in Magnetic bead procedure**. 1. Attachment of adhesive wells onto slides and placement on magnetic workstation. 2. Addition of thinned sputum with TB Beads into adhesive wells on slides. 3. Capture of TB Beads onto magnets below slides on magnetic workstation.

Adhesive wells were firmly attached to microscope slides according to the manufacturer's instructions. Slides were labelled with the appropriate specimen codes and placed on the magnetic workstation (Figure 2).

One millilitre of prepared thinning solution was added to the 1 ml sputum specimen. The top of the sputum container was closed tightly before thoroughly agitating the solution and sputum and incubating for 15 minutes at room temperature.

One ml of Decontamination Agent, 1.0 ml of Capture Buffer and 2 drops (80 μl) of magnetic beads were added to each capture tube (labelled with appropriate specimen code), and mixed gently before the tubes were transferred to the magnetic workstation.

The thinned sputum was mixed with a vortex mixer and 1 ml transferred to the appropriate capture tube. Tubes were incubated for 10 minutes at room temperature to allow mycobacteria to be captured onto the beads. The entire volume of the resulting suspension was transferred carefully to the appropriate slide well using a sterile Pasteur pipette. After 1-3 minutes for capture of the beads on to the surface of the slide (visible as clearing of the liquid around the magnet), the liquid was removed using a transfer pipette.

The wells were gently filled with distilled water. The water was then removed using a transfer pipette. As much water as possible was removed to shorten the drying time required for the slides. Adhesive wells were removed from the slides using forceps, and the slides allowed to air dry at room temperature in the biological safety cabinet.

Magnetic bead slides were stained with auramine and examined as described for concentrated FM smears.

Slides were stored in a closed slide box for re-checking of discrepant results. Specimens with a discrepancy between the magnetic bead FM result and culture were blindly re-checked by a second reader.

### Examination time

A selection of corresponding direct ZN, magnetic bead and concentrated FM slides were examined for varying lengths of time (0.5, 1, 3 and 5 minutes) and the results obtained at the different time points were recorded. Slides selected were those in which any of the methods were positive, plus a random selection of slides that were negative by all methods. The incremental gain in positive results was calculated at each time point, based on the total number of positives recorded by each method.

### Assessment of hands on time and complexity

The total time required for processing samples according to the magnetic bead protocol, as well as the hands on time, was recorded for 3 batches of between 8-10 specimens. A qualitative assessment of the complexity of the method compared with direct ZN and concentration by centrifugation was made.

### Sample size

A sample size of 288 specimens was powered to detect a 20% difference in sensitivity between magnetic bead-FM and direct ZN, assuming sensitivity of direct ZN to be 50% and magnetic bead to be 70% compared with culture. The estimated prevalence of culture positives in the study population was 20%. A drop-out rate of 15% due to loss of culture results due to no growth or contamination was included in the sample size calculation. It was estimated that the sensitivity of concentrated FM and magnetic bead were similar and the study was not powered to detect a small difference in sensitivity between these methods.

### Data analysis

Statistical tests were performed using Intercooled STATA 8.0 software (Statacorp LP, College Station, TX, USA) and Microsoft Excel 7.0 (Microsoft Corporation). Results were considered significant at p < 0.05.

### Ethical considerations

The study was approved by Makerere University, Department of Medicine, Mulago Hospital and Infectious Disease Institute Ethical Committee. Patient identifiers were removed from sputum specimens prior to transfer to the FIND laboratory. Study data were kept in a secure fashion, with access restricted to study personnel only.

## Results

A total of 303 sputum specimens were included in the study. 19 specimens had contaminated cultures on both solid and liquid culture and were excluded from analysis. One ZN slide broke and could not be read. The smear prepared by magnetic bead FM for one specimen washed off the slide completely during staining and no result was available. 282 specimens had results for all microscopy methods and culture and hence were used in comparison of performance.

A summary of performance of direct ZN, magnetic bead-FM and concentrated FM compared with LJ and MGIT culture is given in Table 1. Performance parameters (sensitivity, specificity, overall accurary and positive and negative predictive values) calculated for direct ZN, concentrated FM and magnetic bead-FM compared with culture are shown in Table 2. Performance parameters were calculated using initial readings for all methods.

**Table 1:** Performance of Ziehl-Neelsen (ZN), Magnetic bead-FM (fluorescence microscopy) and Concentrated FM compared with culture on MGIT 960 and Lowenstein-Jensen medium

		Culture (MGIT + LJ)		
		
		Positive	Negative	Total
**Direct ZN**				

	**Positive**	55	2	57

	**Negative**	52	173	225

	**Total**	107	175	282

				

**Magnetic bead FM**				

	**Positive**	70	20	90

	**Negative**	37	155	192

	**Total**	107	175	282

**Concentrated FM**				

	**Positive**	80	10	89

	**Negative**	27	165	193

	**Total**	107	175	282

**Direct FM**				

	**Positive**	63	1	64

	**Negative**	44	174	218

	**Total**	107	175	282

MGIT, mycobacterial growth indicator tube

LJ, Lowenstein-Jensen medium

FM, fluorescence microscopy

ZN, Ziehl-Neelsen

**Table 2:** Performance parameters of ZN, Magnetic bead FM and concentrated FM compared with culture

	Direct ZN	Magnetic bead-FM	Concentrated FM	Direct FM*
**Sensitivity, %** **(95% CI)**	51.4% (41.5 - 61.2)	65.4% (55.6 - 74.4)	74.8% (65.4 - 82.7)	58.9% (49.0 - 68.3)

**Specificity, %** **(95% CI)**	98.9% (95.9 - 99.9)	88.6% (82.9 - 92.9)	94.3% (89.7 - 97.2)	99.4% (96.9 - 100.0)

**Overall accuracy, %** **(95% CI)**	80.9% (75.8 - 85.3)	79.8% (74.6 - 84.3)	86.9% (82.4 - 90.6)	84.0% (79.2 - 88.1)

**PPV, %** **(95% CI)**	96.5% (87.9 - 99.6)	77.8% (67.8 - 85.9)	88.9% (80.5 - 94.5)	98.4% (91.6 - 100.0)

**NPV, %** **(95% CI)**	76.9% (70.8 - 82.2)	80.7% (74.4 - 86.1)	85.9% (80.2 - 90.5)	78.9% (73.9 - 84.9)

* Direct FM was performed in the routine microbiology laboratory, Mulago Hospital

CI, confidence interval

PPV, positive predictive value

NPV, negative predictive value

ZN, Ziehl-Neelsen

FM, fluorescence microscopy

Concentrated FM had significantly higher sensitivity than direct ZN (p < 0.001), magnetic bead-FM (p = 0.035) and direct FM (p = 0.0002). The sensitivity of magnetic bead was significantly higher than direct ZN (p < 0.001) but not significantly higher compared with direct FM (p = 0.210).

The specificity of magnetic bead FM and concentrated FM was significantly lower than direct ZN (p < 0.001 and p = 0.039 respectively) and direct FM (p < 0.001 and p = 0.004 respectively). There was no significant difference in specificity between magnetic bead FM and concentrated FM (p = 0.076).

Of 20 specimens that were false positive by magnetic bead-FM, 2 were also positive by concentrated FM (all were negative by direct ZN). 17/20 magnetic bead-FM false positive results were very low positive results, with 14 slides having between 1 and 3 AFBs reported per 40 fields. However, three slides were reported as 3+ positive. 12/20 false positive TB-bead slides were confirmed as smear-positive by blinded re-checking of the magnetic bead-FM slides; the remaining 8 slides were reported as smear- negative by the second reader (Table 3). If the three strongly smear positive first readings were removed as potential mislabelling errors the specificity of the magnetic bead FM would increase to 90.1% (155/172).

**Table 3:** Discrepant TB-bead FM results compared with culture: results of direct ZN and concentrated FM, and blinded re-checking of Magnetic bead results

Lab No	Direct ZN	Conc FM	Magnetic bead-FM	Magnetic bead re-read*
**Magnetic bead false positives (compared to culture)**				

261	Negative	Negative	3+	3+

206	Negative	Negative	1 AFB/40F	Negative

099	Negative	Negative	1 AFB/40F	Negative

259	Negative	Negative	3+	3+

296	Negative	Negative	8 AFB/40F	Negative

082	Negative	1+	13 AFB/40F	Negative

276	Negative	Negative	2 AFB/40F	Negative

203	Negative	Negative	1 AFB/40F	Negative

287	Negative	Negative	1 AFB/40F	Negative

262	Negative	Negative	1 AFB/40F	3+

141	Negative	Negative	1 AFB/40F	2 AFB/40F

256	Negative	Negative	2 AFB/40F	13 AFB/40F

096	Negative	Negative	1 AFB/40F	8 AFB/40F

180	Negative	Negative	3 AFB/40F	1 AFB/40F

131	Negative	Negative	1 AFB/40F	1 AFB/40F

299	Negative	Negative	3+	1+

302	Negative	Negative	2 AFB/40F	3+

211	Negative	1+	4 AFB/40F	Negative

267	Negative	Negative	2 AFB/40F	3 AFB/40F

299	Negative	Negative	2 AFB/40F	1+

Magnetic bead false negatives compared to culture (with one or more positive result by conc FM or ZN)**				

283	5 AFB/40F	2+	Negative	1+

280	2 AFB/40F	2+	Negative	2 AFB/40F

260	1+	2+	Negative	1+

258	6 AFB/40F	3+	Negative	Negative

151	1+	3+	Negative	Negative

084	1+	1+	Negative	Negative

294	Negative	15 AFB/40F	Negative	Negative

292	Negative	5 AFB/40F	Negative	3 AFB/40F

210	Negative	2 AFB/40F	Negative	Negative

157	Negative	8 AFB/40F	Negative	Negative

034	Negative	5 AFB/40F	Negative	Negative

053	Negative	1+	Negative	Negative

050	Negative	3+	Negative	3 AFB/40F

011	3 AFB/40F	Negative	Negative	4 AFB/40F

* Slides were blindly re-checked

**An additional 23 specimens had false negative results by all three microscopy methods and re-checking was not performed.

37 specimens gave TB-bead false negative results compared with culture. Of these 23 were also negative by concentrated FM and direct ZN and were not studied further. Magnetic bead slides were re-checked for the remaining 14 false negatives and results are shown in Table 3. Resolved performance parameters of the magnetic bead prototype method are given in Table 4, after blinded re-reading of discrepant magnetic bead results.

**Table 4:** Performance parameters of magnetic bead FM following blinded re-checking of discrepant results, compared with culture on MGIT 960 and Lowenstein-Jensen medium

		Culture (MGIT + LJ)		
		
		Positive	Negative	Total
**Magnetic bead FM (with blinded re-checking of discrepants)**				

	**Positive**	76	12	88

	**Negative**	31	163	194

	**Total**	107	175	282

**Sensitivity, %** **(95% CI)**		**71.0%** **(61.5 - 79.4)**		

**Specificity, %** **(95% CI)**		**93.1%** **(88.3 - 96.4)**		

**Overall accuracy, %** **(95% CI)**		**84.8%** **(80.0-88.7)**		

**PPV, %** **(95% CI)**		**86.4%** **(77.4-92.8)**		

**NPV, %** **(95% CI)**		**84.0%** **(80.0-88.7)**		

CI, confidence interval

PPV, positive predictive value

NPV, negative predictive value

ZN, Ziehl-Neelsen

FM, fluorescence microscopy

### Examination time

Results obtained by the three methods with varying examination times are summarized in Table 5. Concentrated FM had the highest sensitivity per time period, which was most marked when slides were only examined for a short time (30 seconds). The number of positive slides identified with concentrated FM after 30 seconds reading time was twice the number identified by Magnetic bead in the same time period. After 1 minute examination time, sensitivity of Magnetic bead had improved, while direct ZN performed poorly overall at all time points. There was no advantage in reading concentrated FM slides for more than 3 minutes, since no additional positive results were obtained. With Magnetic bead slides, however, additional positive slides were found when reading for between 3 and 5 minutes. All additional positives for concentrated FM between 1 and 3 minutes and Magnetic bead between 3 and 5 minutes were very low positives (1 or 2 AFBs per 40 fields). Of 6 Magnetic bead slides that became positive only after more than 3 minutes examination, 2 were true positives and 4 were false positives.

**Table 5:** Sensitivity of direct ZN, concentrated FM and Magnetic bead-FM, as a function of reading time

Reading time	Cumulative No. positive slides	New positive slides	Yield of new positives/total positives* (%)
*Direct ZN*			

30 s	4	4	66.7%

1 min	4	0	0%

3 min	4	0	0%

5 min	6	2	33.3%

*Concentrated FM*			

30 s	12	12	63.2%

1 min	14	2	10.5%

3 min	19	5	26.3%

5 min	19	0	0%

*Magnetic bead*			

30 s	6	6	35.3%

1 min	11	5	29.4%

3 min	13	2	10.5%

5 min	17	4	23.5%

*total number of slides positive by each method after 5 minutes examination time.

### Assessment of hands on time and complexity

Concentrated FM and Magnetic bead-FM were both substantially more complex than direct ZN, having more steps in the procedure, requiring additional equipment, and taking longer to perform (Table 6). Concentrated FM and Magnetic bead-FM had the same number of steps in the procedure, and had approximately the same hands-on time and total time. Examination times were excluded from this estimation, as was time required to prepare solutions and stains for all methods.

**Table 6:** Comparison of complexity, hands on and total time involved in performing direct ZN, Magnetic bead and concentrated FM.

	Direct ZN	Concentrated FM	Magnetic bead-FM
	1. Label slides	1. Label slides	1. Label slides

	2. Transfer sputum using applicator and prepare smear	2. Transfer sputum to centrifuge tube	2. Attach adhesive wells

	3. Air dry	3. Add decontamination agent and mix	3. Add bleach solution to sputum

	4. Heat fix	4. Incubate	4. Incubate

	5. Transfer slides to staining rack and add stain	5. Add buffer to 50 ml mark	5. Add capture buffer, Magnetic bead and decon agent to tubes

	6. Rinse with water and drain	6. Load centrifuge	6. Add thinned sputum to capture tube

	7. Add decoloriser	7. Centrifuge	7. Incubate

	8. Rinse with water and drain	8. Unload, pour off supernatant and suspend pellet	8. Add suspension to wells, wash and remove water from wells

	9. Add counterstain	9. Make smears	9. Remove wells

	10. Drain and rinse with water	10. Air dry	10. Air dry

	11. Blot back of slides with tissue	11. Heat fix	11. Heat fix

	12. Air dry	12. Transfer slides to staining rack and add stain	12. Transfer slides to staining rack and add stain

	13. Examine slides under microscope	13. Rinse with water and drain	13. Rinse with water and drain

		14. Add decoloriser	14. Add decoloriser

		15. Rinse with water and drain	15. Rinse with water and drain

		16. Add counterstain	16. Add counterstain

		17. Drain and rinse with water	17. Drain and rinse with water

		18. Blot back of slides with tissue	18. Blot back of slides with tissue

		19. Air dry	19. Air dry

		20. Examine slides under microscope	20. Examine slides under microscope

Hands on time (mins)*	34.0	48.5	48.7

Total time (mins)*	74.6	146.6	139.5

* Hands-on time and total time were estimated based on 3 batches of slides prepared (8-10 slides per batch). These times excluded examination times.

Technologists reported that the magnetic bead FM slides were easier to read than direct ZN and comparable to concentrated auramine. It was an advantage for reading slides that the area where AFB had been concentrated using the magnetic workstation was clearly defined and visible which helped with placing the slide on the microscope stage. Very highly positive slides were found to be excessively bright due to high concentration of fluorescent stain.

## Discussion

This early prototype magnetic bead processing method involved a similar level of complexity and laboratory infrastructure to concentration using decontamination and centrifugation, and both methods were significantly more time-consuming than performing direct smear preparation. The two concentration methods investigated (magnetic bead FM and concentrated FM) both had higher sensitivity than direct ZN, however the specificity was reduced for both methods. Reduced specificity of concentrated microscopy compared with direct smears has been reported previously [10,11]. In their study of HIV-infected pulmonary TB suspects in Uganda, Cattamanchi *et al *[10] reported significantly lower specificity of concentrated ZN smear compared with direct ZN (92% and 99% respectively). However Steingart *et al *[3] in a systematic review found that all but one study [12] showed similar specificity in direct and concentrated smears. After excluding results from that outlying study, they reported a mean specificity of 98% (92-100%) for direct smear and 98% (91-100%) for concentrated smears. They speculated that the low specificity found in the excluded study may be due to contamination of culture media or inclusion of patients on TB treatment.

The three methods using fluorescence staining (magnetic bead FM, direct FM and concentrated FM) all had higher sensitivity than direct ZN, as has been widely reported in other studies [4]. The improvement in sensitivity of the magnetic bead FM compared with direct ZN may, however, have been in large part due to the use of auramine staining and not just the concentration effect of the magnetic bead procedure, since the sensitivity of magnetic bead was not significantly higher than direct FM, despite the fact that direct FM was performed in a busy routine laboratory.

Many of the false positive magnetic bead FM results were very low positives, presumably due to fluorescent staining of debris. However the technologists who performed the testing were experienced with FM prior to start of study and thus would be expected to differentiate fluorescent debris from acid-fast bacilli. A large proportion of the false positive results had 3 or less AFBs observed per slide. Therefore increasing the cut-off for reporting a positive result could be considered as a means of improving the specificity of the magnetic bead method. Furthermore, slides with very low numbers of AFBs could be re-checked at higher magnification. Longer examination times (more than 3 minutes) also contributed to the reporting of false positive results. In some cases the background of magnetic bead FM slides was high, which may have hindered examination. Upon re-checking of results by a second microscopist, some false positive results were recorded as negative, which is not unexpected given that most false positives had very low numbers of bacilli. The small number of highly false positive results, which were confirmed on re-checking, could not be readily explained and may have resulted from a labeling error during the coding of slides.

Since splitting of the sputum specimens was required prior to allocating sputum to magnetic bead processing or decontamination and centrifugation for concentrated auramine and culture, it is possible that low numbers of bacilli were present in one portion and not the other. Some of the specimens giving false positive results were contaminated on MGIT and therefore relied on a negative LJ result alone for comparison. Furthermore, culture is not a perfect gold standard method. It is possible that a low number of bacilli were present in the portion of specimen for culture and LJ was falsely negative. In addition, although all specimens in this study were reported as being from patients who were not on TB treatment, it may be possible that some patients may in fact have received TB treatment, and that cultures may be negative due to effect of anti-tuberculous drugs. Two of the magnetic bead FM false positive specimens were also positive on concentrated FM.

All manipulations for this study were carried out in a biological safety cabinet, including magnetic bead processing (excluding reagent preparation). Although biosafety issues were not formally assessed in this study, it is recommended that this current version of the procedure should be performed in a BSC since extensive manipulations of potentially infectious suspensions is required. However, the bleach treatment of sputum, which is part of the magnetic bead procedure, may reduce the infectiousness of the sample at an early stage, although the concentration may not be sufficient for complete kill. This aspect should be further investigated.

## Conclusion

Simple methods to improve the performance of smear microscopy in low and middle income countries are urgently needed. In this study a prototype magnetic bead FM method and concentrated FM had higher sensitivity than direct ZN, however in both methods the specificity was significantly lower than direct ZN and direct FM. Sensitivity of the prototype magnetic bead FM was not significantly higher than direct FM and the prototype magnetic bead procedure was much more complex to perform than direct smear preparation, and was similar to concentration by centrifugation. Some magnetic bead FM false positive results were not easily explained and should be further investigated. Improvements in specificity and biosafety, and simplification of the magnetic bead procedure will be required before this method would be suitable for implementation in microscopy laboratories in developing countries. If the sensitivity of the magnetic bead FM could be improved in future versions of the technology this may offer a viable alternative to centrifugation.

## Competing interests

HA, PJA, GL, BN and MP are employed by the Foundation for Innovative New Diagnostics (FIND). MJ and YM declare they have no competing interests.

SW is employed by Microsens MedTech Ltd. Microsens Medtech Ltd. was involved in provision of reagents and training of laboratory personnel. They had no role in performance of the study, data analysis or the decision to publish, but reviewed the manuscript prior to publication.

## Authors' contributions

HA conceived the study, participated in study design, coordinated the project, performed data analysis and drafted the manuscript. PA, GL and NB carried out laboratory testing and participated in study coordination. MP conceived the study, participated in study design and critically reviewed the manuscript. YM and MJ participated in study design and critically reviewed the manuscript. SW provided training and technical support and critically reviewed the manuscript. All authors read and approved the final manuscript.

## Pre-publication history

The pre-publication history for this paper can be accessed here:

http://www.biomedcentral.com/1471-2334/11/125/prepub
